# Cortex-Wide Neuron Activation after Traumatic Brain Injury in Mice

**DOI:** 10.1523/ENEURO.0020-26.2026

**Published:** 2026-05-22

**Authors:** Alexa Tierno, Aidan Baraban, Robert F. Hunt

**Affiliations:** ^1^Department of Anatomy & Neurobiology, University of California, Irvine, California 92697; ^2^Epilepsy Research Center, University of California, Irvine, California 92697

**Keywords:** c-Fos, immediate early genes, neocortex, neuron activation, traumatic brain injury

## Abstract

Following a traumatic brain injury (TBI), the neocortex undergoes time-dependent cellular responses including immediate tissue deformation, enhanced excitability, and elevated expression of immediate early genes. However, the spatial extent of early neuronal activity after a focal injury remains unclear. Here we use targeted recombination in active populations reporter mice of both sexes to identify neurons activated in the acute phase following a controlled cortical impact injury applied to the somatosensory neocortex. We find widespread cell activation across large portions of the cortex that extends beyond the astrocytic and microglial responses marking the injury site. Activated cells are predominantly neurons, and few cells colabel with GFAP or IBA1. Our findings reveal that even focal injury engages cortical circuits across large portions of the injured brain, highlighting the importance of considering cortex-wide neuronal dynamics in the early postinjury period and their potential impact on network excitability, remodeling, and recovery.

## Significance Statement

Traumatic brain injury leads to increased expression of immediate early genes, which are commonly used as surrogate markers of cell activity, but the spatial extent of early neuron activation remains unresolved. Here, using a transgenic reporter mouse line, we show that focal contusion injury leads to an activation of neurons across large portions of the ipsilateral neocortex that extends well beyond the lesion area. These findings show the whole injured hemisphere becomes engaged in the early response to focal injury, suggesting cortex-wide neuronal dynamics in the early postinjury period may influence network excitability and recovery.

## Introduction

Traumatic brain injury (TBI) can lead to life-long physical, emotional, and cognitive disabilities. The initial response to injury involves a wide range of modifications to brain structure and function, including tissue deformation, excitotoxicity, cell death, neuroinflammation, metabolic dysfunction, and progressive neural circuit remodeling (Hunt et al., 2013). One of the most robust indicators of acute brain damage is the activation of immediate early genes (IEGs; Herrera and Robertson, 1996). IEGs, such as *fos* and *Arc*, are genes that are expressed at low levels in quiescent cells but become rapidly activated by cellular stimulation (Sheng and Greenberg, 1990; Lanahan and Worley, 1998). As a result, IEG expression is frequently used as a marker of neuronal activity in studies attempting to link brain circuit function to behavior (Guzowski et al., 2005; DeNardo and Luo, 2017; Nambu et al., 2022) and in experimental models of epilepsy (Dragunow and Robertson, 1987; Lauterborn et al., 1996), ischemia (Kiessling and Gass, 1993), or TBI in rodent and human (Dragunow and Robertson, 1988; Raghupathi et al., 1995b; Dutcher et al., 1999).

In experimental models, TBI is almost immediately followed by increased expression of IEGs in the neocortex and hippocampus, as determined by histological analysis. Among studies of comparable injury severities, some have shown IEG activation focally restricted to areas near the lesion (Dragunow and Robertson, 1988; Dragunow et al., 1990; Förstner and Knöll, 2020) whereas other studies have documented more widespread patterns of IEG activation involving c-*fos* expression in one or both hemispheres (Phillips and Belardo, 1992; Yang et al., 1994, 1995; Belluardo et al., 1995; Raghupathi et al., 1995a; Abrous et al., 1999). Although more commonly used as an activity marker, IEGs have been repeatedly shown to participate in molecular mechanisms of plasticity underlying behavior, such as memory (Nambu et al., 2022). This raises the possibility that acute IEG expression may mark sites of future circuit remodeling, which could involve areas across the brain beyond the site of injury (Harris et al., 2016; Frankowski et al., 2022). Therefore, a deeper understanding of acute neuron activation in response to TBI is important, because it has the potential to reveal the earliest evidence that a neuron will undergo synaptic changes. Here we used a transgenic mouse line, called targeted recombination in active populations (TRAP), to label all of the cell populations that were activated by a controlled cortical impact (CCI) injury. This approach revealed extensive neuron activation across the neocortex, hippocampus, and select subcortical brain areas.

## Materials and Methods

### Animals

All animal procedures were performed under Institutional Animal Care and Use Committee approval by the University Laboratory Animal Resources and adhered to National Institutes of Health Guidelines for the Care and Use of Laboratory Animals. Experiments were performed on adult mice of both sexes maintained in standard housing conditions on a 12 h light/dark cycle with food and water provided *ad libitum*. Homozygous Fos^tm2.1(icre/ERT2)Luo/J^ (or TRAP2) mice (Jax Stock Number 030323) were crossed with homozygous Cg-Gt(ROSA)26Sor^tm14(CAG-tdTomato)Hze/J^ (or Ai14) reporter mice (Jax Stock Number 007914) to produce hemizygous offspring, as done previously (DeNardo et al., 2019).

### Brain injury

CCI injury was performed on male and female mice at Postnatal Day (P)60 (Frankowski et al., 2022). Briefly, mice were anesthetized by 2% isoflurane inhalation and placed on a custom stereotaxic frame. The skull was exposed by a midline incision, and a 4–5 mm craniotomy was made ∼1 mm from the sagittal suture and centered between the lambda and bregma. The skull cap was removed using fine forceps. The CCI device consisted of a computer-controlled, pneumatically driven impactor fitted with a beveled stainless-steel tip 3 mm in diameter (Precision Systems and Instrumentation; TBI-0310). Brain injury was delivered to the neocortex using the following parameters: depth, 1.0 mm; velocity, 3.5 m/s; and dwell time, 500 ms. The incision was sutured without replacing the skull cap, and animals were allowed to recover on a heating pad. All animals, except a portion of controls, received buprenorphine hydrochloride (Buprenex; 0.05 mg/kg, i.p.) at the time of surgery and 12 h later. Control mice were anesthetized by 2% isoflurane and remained anesthetized for ∼30 min (or about the duration of a CCI surgery) and allowed to recover on a heating pad.

### Tamoxifen preparation and delivery

Tamoxifen (Sigma-Aldrich, catalog #5648) was dissolved at 20 mg/ml in corn oil (Sigma-Aldrich, catalog #C8267) by shaking at 37°C overnight. Immediately prior to CCI surgery, all mice were injected intraperitoneally with 75 mg/kg of tamoxifen. Similar studies using tamoxifen injections have been performed in a contusion model of spinal cord injury (Brown et al., 2022).

### Immunohistochemistry

Mice were transcardially perfused with 4% paraformaldehyde (v/v), and free-floating vibratome sections (50 μm) were processed using standard immunostaining procedures. Primary antibodies were chicken anti-NEUN (1:1,000; Millipore, catalog #Abn91, RRID:AB_11205760), rabbit anti-DsRed (1:1,000; Takara Bio, catalog #632496, RRID:AB_10013483), mouse anti-GFAP (1:500; Millipore, catalog #MAB3402, RRID:AB_94844), and rabbit anti-IBA1 (1:1,000; FUJIFILM Wako Pure Chemical, catalog #019-19741, RRID:AB_839504). For secondary antibodies (1:1,000; Life Technologies), we used Alexa Fluor 488-conjugated goat antibody to mouse IgG (catalog #A-11029, RRID:AB_2534088), goat anti-chicken (catalog #A-11039, RRID:AB_2534096), Alexa Fluor 546-conjugated goat antibody to rabbit (catalog #A-11035, RRID:AB_2534093), and Alexa Fluor 647-conjugated goat antibody to rabbit (catalog #A-21244, RRID:AB_2535812). Blocking buffer consisted of 10% normal goat serum, 1% BSA, and 0.1% Triton X-100 in PBS; primary antibodies were incubated overnight at 4°C and secondary antibodies incubated at room temperature for 2 h. As expression of tdTomato was already strong in activated cells, we did not perform immunostaining against tdTomato in most experiments. Sections were then mounted on charged slides (Superfrost plus, Thermo Fisher Scientific) with Fluoromount-G that contained DAPI. Epifluorescent images were obtained using a Leica DM6 microscope with the Leica LAS X software. Confocal images were obtained with an Olympus FV3000 laser scanning microscope. Brightness and contrast were adjusted manually using FIJI (ImageJ) and applied to the entire image.

### Volumetric analysis

Coronal brain sections (50 μm thick, 300 μm apart) that comprised the entire rostral to caudal extent of the lesion were mounted on slides and coverslipped with Fluoromount-G containing DAPI. Sections were imaged at 5× magnification using a Leica DM6 microscope. Eight sections were analyzed per animal, and the percentage of the ipsilateral cortex remaining was calculated by outlining the borders of the neocortex in both hemispheres using FIJI (ImageJ) and calculating the ratio of ipsilateral to contralateral cortex volume (Frankowski et al., 2019; Zhu et al., 2019). The neocortex was defined as the region between the dorsal aspect of the corpus callosum and the pial surface. Regions of the cortical subplate (e.g., amygdala, endopiriform nucleus) were excluded from analysis.

### Immunostaining analysis

Fluorescently labeled sections (50 μm, 300 μm apart) were imaged at 5–40× using a Leica DM6 microscope or Olympus FV3000 confocal microscope and quantified using FIJI (ImageJ). For quantification of the percentage area, the ipsilateral and contralateral cortical area was outlined, and the percentage area above the fluorescence threshold was measured using ImageJ according to a previous protocol (Jensen, 2013; Frankowski et al., 2021). The same settings were used for all sections. For measurements of the dorsal neocortex, a region of interest was drawn 2 mm lateral to the edge of the cavity in the ipsilateral neocortex, and 16 consecutive sections (every sixth coronal section, 300 μm apart) were quantified and averaged per animal. For paraventricular nucleus of the thalamus (PVT) and somatosensory neocortex analyses, three consecutive brain sections 300 μm apart were analyzed through the PVT or somatosensory cortex per animal. For colocalization analysis, a region of interest was randomly selected lateral to the contusion cavity in the ipsilateral neocortex. All cells that expressed Ai14 and/or NEUN, GFAP, and IBA1 were counted at the lesion epicenter and ±300 μm from the lesion epicenter, and the values were averaged to obtain a mean percent cell colocalization per animal.

### Statistical analysis

All statistical tests were performed with GraphPad Prism 10. Data were compared by two-tailed Student's *t* test, two-way ANOVA followed by a Tukey post hoc test for multiple comparisons, two-way repeated–measures ANOVA followed by Bonferroni’s post hoc test or Fisher's exact test. Normality was assessed in GraphPad at the time of analysis. Data are expressed as mean ± SEM, *N* = animals, and significance was set at *p* < 0.05.

## Results

**Figure 1.**
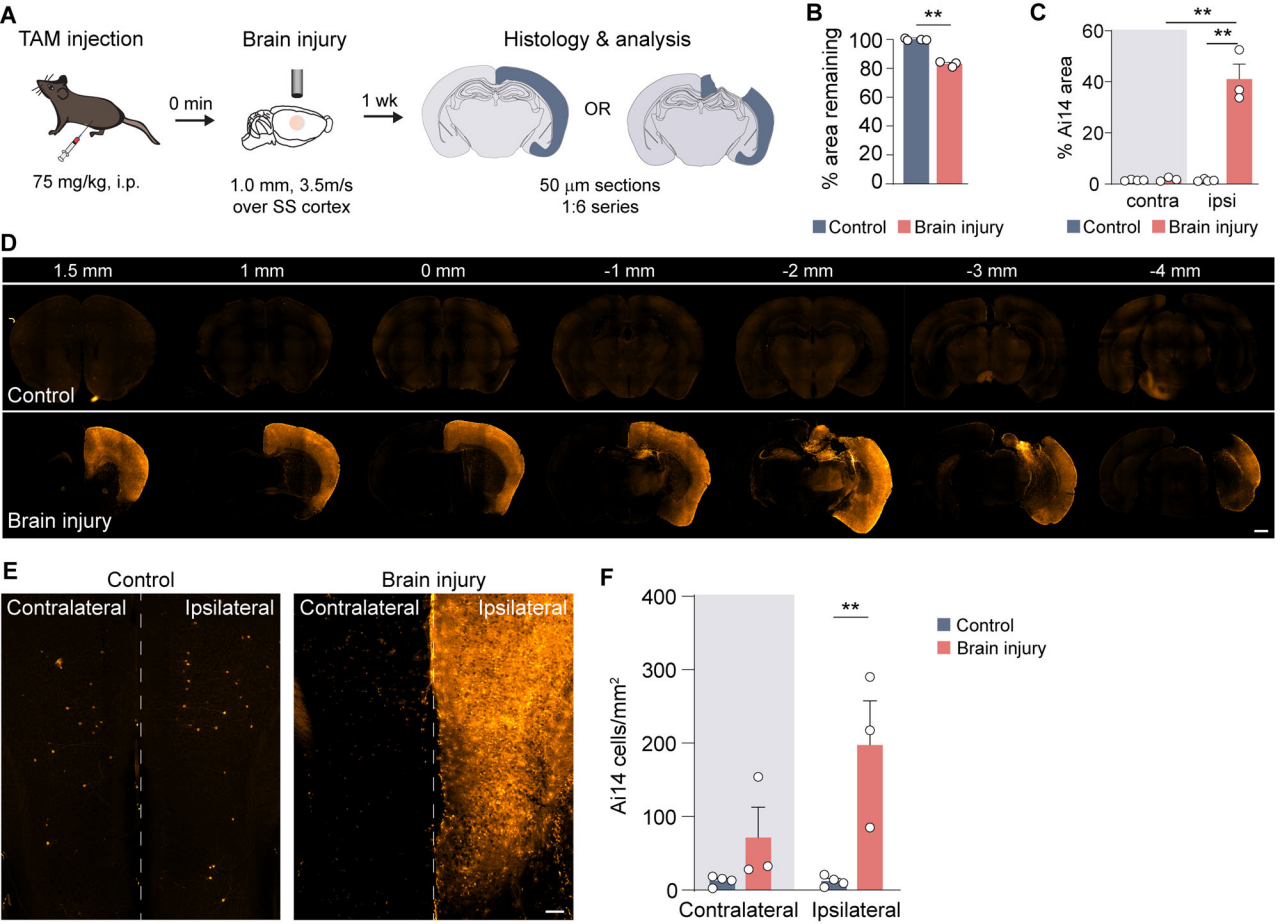
Hemisphere-wide cell activation following TBI. ***A***, Experimental design schematic. ***B***, Quantification of the percentage tissue remaining ipsilateral to the injury in control and brain-injured mice. **p = 2.3 × 10^−3^, control versus TBI; two-tailed *t* test, *N* = 3–4 mice per group. ***C***, Quantification of the percentage area of Ai14 expression in contralateral or ipsilateral hemispheres. **p = 2.27 × 10^−6^, control versus brain injury; **p = 4.59 × 10^−6^, brain injury ipsilateral versus contralateral; two-way ANOVA with Tukey's post hoc analysis, *N* = 3–4 mice per group. ***D***, Coronal brain sections of Ai14 reporter expression every 300 μm through the brain of representative control and brain-injured animals. Scale bar, 1 mm. ***E***, Ai14 reporter expression in the prelimbic area of PFC. Scale bar, 100 µm. ***F***, Quantification of Ai14 + cell density in contralateral and ipsilateral hemisphere of PFC in control and brain-injured animals. **p = 7.3 × 10^−3^, ipsilateral control versus brain injury; two-way ANOVA with Tukey's post hoc analysis, *N* = 3–4 mice per group.

To visualize early neuron activation in response to TBI, we used TRAP, which allows for permanent genetic labeling by a specific experience (DeNardo et al., 2019). We crossed a TRAP2 Cre-driver mouse line with mice expressing Ai14 as a reporter and delivered a 1.0 mm depth CCI injury over the somatosensory cortex at P60 (Fig. 1*A*). Control mice were anesthetized but did not receive a brain injury. Mice were administered tamoxifen immediately prior to brain injury to label cells activated within a recombination window up to 36 h after the time of brain injury (Guenthner et al., 2013; Onishi et al., 2024). Upon visual inspection of the lesion site after impact, the dura was found to be intact in the brain-injured animals.

At 7 d after TBI, the lesion consisted of a cavity extending through the thickness of the neocortex and included substantial distortion of the hippocampus (*N* = 3 mice). Uninjured controls showed no overt cortical lesion in any animal examined (Fig. 1). Brain-injured animals had ∼20% decrease in cortical volume ipsilateral to the injury, as compared with uninjured controls (Fig. 1*B*; control, 100 ± 0.3%; TBI, 83 ± 1.1%; *p* = 0.002; two-tailed *t* test). This is nearly identical to prior observations using similar impact parameters (Frankowski et al., 2019; Zhu et al., 2019).

Next, we examined neuronal activation after TBI by quantifying Ai14 reporter expression, which labels cells that were active around the time of injury, in the ipsilateral and contralateral neocortex. In uninjured controls, Ai14 expression was very low, as expected (DeNardo et al., 2019), with Ai14-labeled cells found sparsely distributed throughout the brain (Fig. 1*C*,*D*). We found a significant increase in the percentage of Ai14 area in the ipsilateral, but not contralateral, hemisphere in mice that received a TBI, as compared with controls (Fig. 1*C*,*D*). In brain-injured animals, Ai14^+^ cells were found throughout the ipsilateral neocortex. We also noted substantial Ai14^+^ expression in the amygdala, internal capsule, cerebral peduncle, and lateral thalamic nuclei known to interact with the somatosensory neocortex, including the reticular thalamus and ventrobasal complex. In the hippocampus, moderate Ai14^+^ expression was observed in dentate gyrus granule cells of the contralateral hemisphere at the injury epicenter of all brain-injured animals. Ai14 expression was sparse or absent in these brain areas of control animals. These results suggest brain injury produces an early, but selective, activation of multiple cortical and subcortical brain areas.

Having previously observed brain-wide reorganization of interneuron circuitry in the prefrontal cortex (PFC) (Frankowski et al., 2022), which interacts with the dorsal hippocampus but is not directly damaged by CCI injury (Hall et al., 2005, 2008; Frankowski et al., 2022), we asked whether PFC is activated by the acute injury. Quantification of Ai14^+^ cells in the prelimbic area of PFC revealed a significant increase in cell activation in the injured hemisphere (Fig. 1*E*,*F*).

**Figure 2.**
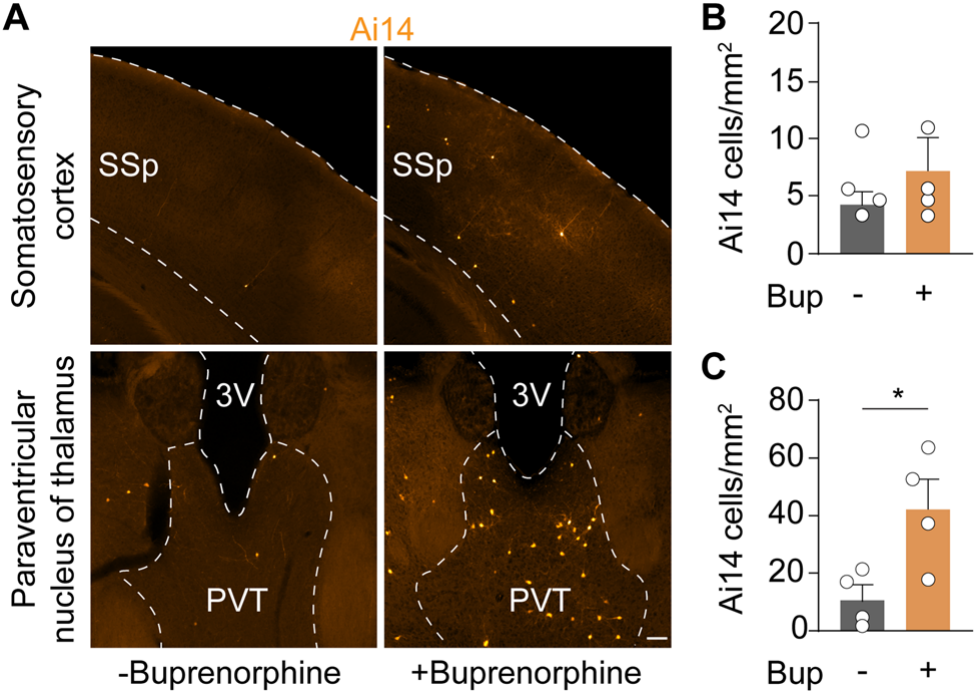
Buprenorphine activates a small number of cells. ***A***, Ai14 expression in the somatosensory neocortex (top) and PVT (bottom) of mice with and without a single administration of 0.05 mg/kg buprenorphine. Scale bar, 100 µm. ***B***, ***C***, Quantification of Ai14 expression in the somatosensory cortex (***B***) and PVT (***C***). PVT: *p = 4.2 × 10^−2^; −Bup versus + Bup; *N* = 4 mice per group; two-tailed *t* test. SSp, primary somatosensory cortex; 3V, third ventricle.

During surgery, animals were administered buprenorphine, a synthetic opioid analgesic that acts as a partial mixed opioid agonist at the μ-receptor (Kumar et al., 2024) and has potential to activate neurons independent from TBI. To test the effect of buprenorphine on neuron activation, we examined Ai14 expression in the somatosensory neocortex and PVT, a brain area that exhibits opioid receptor-dependent modulation of firing (Hou et al., 2023), in mice with and without a buprenorphine injection. These experiments were only performed in uninjured control animals, because it is not ethical to withhold analgesics from brain-injured animals. In both brain areas, we observed a significant increase in Ai14-labeled cells following buprenorphine administration, as compared with animals that did not receive buprenorphine (Fig. 2). However, the increase in Ai14 expression was modest. We conclude that the increase in cell activation after TBI is much more likely to be driven by TBI than a heightened response to analgesics.

**Figure 3.**
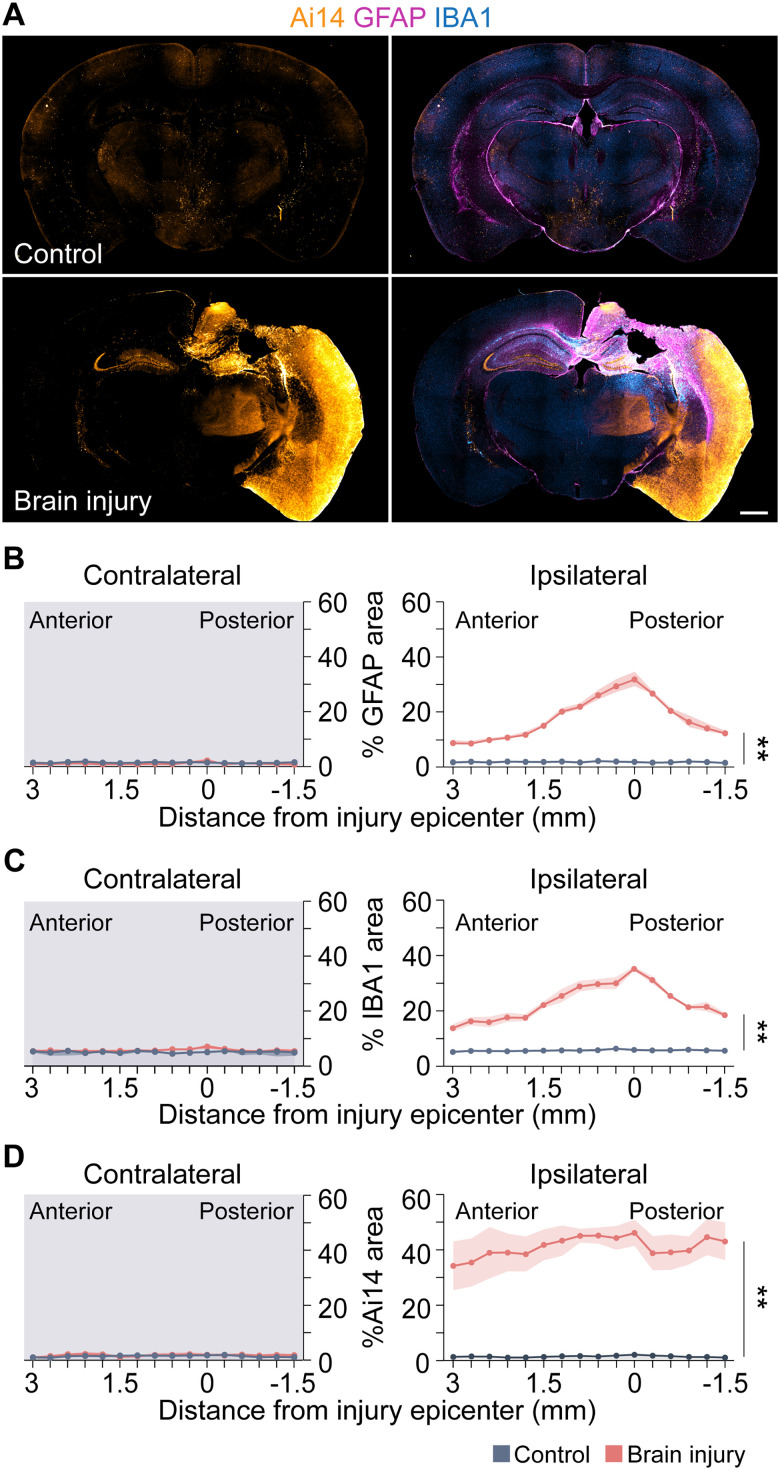
Cell activation extends beyond the glia scar. ***A***, Coronal brain sections of Ai14 (orange), GFAP (magenta), and IBA1 (blue) at the injury epicenter of a control and brain-injured mouse. Representative animals are from *N* = 4 controls and 3 TBI animals. Scale bar, 100 µm. ***B–D***, Quantification of GFAP (***B***), IBA1 (***C***), or Ai14 (***D***) expression in 16 sequential brain sections of the neocortex along the anterior–posterior (AP) axis contralateral (left) and ipsilateral (right) to the injury (GFAP, **p = 3.7 × 10^−9^, control vs brain injury; IBA1, **p = 3.3 × 10^−6^, control vs brain injury; Ai14, **p = 4.6 × 10^−4^, control vs brain injury; two-way rmANOVA with Bonferroni’s post hoc analysis).

To label the extent of the lesion, we examined glial responses in the neocortex (Fig. 3*A*). To do this, we performed an immunostaining analysis at 7 d after injury for glial fibrillary acidic protein (GFAP), a marker of astrocytes, and ionized calcium-binding adaptor molecule 1 (IBA1), a marker of activated microglia. Comparisons were made between age-matched uninjured controls and brain-injured animals. In all brain-injured animals, the impact site could be clearly identified by a dense pattern of GFAP and IBA1 staining ipsilateral to the injury. No overt neocortical lesion was observed in any uninjured control animal. Analysis of the glial responses every 300 μm along the anterior–posterior axis revealed GFAP and IBA1 immunostaining was most robust at the lesion site, decreased with distance from the injury epicenter and was significantly higher in brain-injured mice compared with controls (Fig. 3*B*,*C*). In contralateral hemisphere, we did not observe a difference between groups, consistent with the relatively focal lesion produced by CCI injury. This pattern of neuroinflammation is qualitatively similar with prior studies demonstrating increases in glial responses following 1.0 mm impact depths (Zhu et al., 2019).

Analysis of Ai14 expression every 300 μm along the anterior–posterior axis revealed intense immunostaining throughout the ipsilateral hemisphere that did not decrease with distance from the injury epicenter (Fig. 3*D*). In these animals, the area of cell activation did not peak at the lesion area and taper with distance from the injury epicenter, as seen with GFAP and IBA1 expression. Instead, we observed elevated Ai14 immunostaining throughout most of the ipsilateral neocortex. Taken together, these results suggest there is broad cell activation in the ipsilateral hemisphere in the early period after a contusion injury that extends beyond the area directly damaged by the injury.

**Figure 4.**
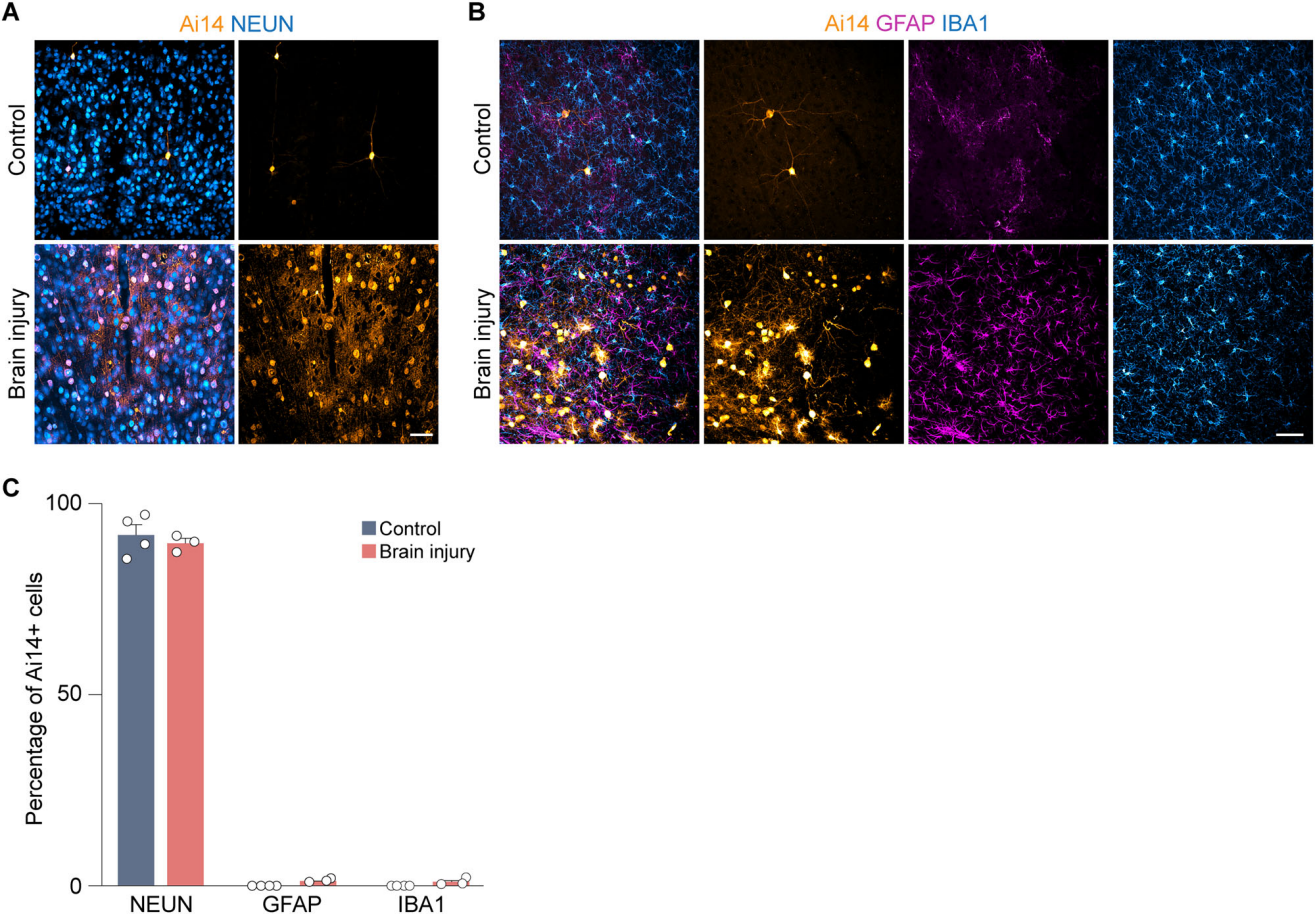
Ai14^+^ cells are putative neurons. ***A***, Coronal brain sections of Ai14 (orange) and NEUN (blue) near the injury site in the neocortex of a control and brain-injured mouse. ***B***, Coronal brain sections of Ai14 (orange), GFAP (magenta), and IBA1 (blue) at the injury site in the neocortex of a control and brain-injured mouse. Scale bar, 50 µm. ***C***, Quantification of Ai14^+^ cells that colabel for NEUN, GFAP, or IBA1.

Nearly all Ai14^+^ cells were morphologically identified as neurons, consistent with prior studies reporting that TRAP preferentially labels neurons (Guenthner et al., 2013). This was confirmed by colabeling with NEUN, and no difference was detected between groups (control, 92 ± 3%; TBI, 90 ± 1%; *p* = 0.49; two-tailed *t* test; *N* = 3–4 mice per group; Fig. 4*A*,*C*). To test whether glia were also labeled after TBI, we quantified Ai14^+^ cell colocalization with GFAP and IBA1 in the neocortex just lateral to the injury site. In controls, none of the Ai14^+^ cells expressed GFAP or IBA1 (Fig. 4*B*,*C*). In brain-injured animals, only ∼2% of Ai14^+^ cells expressed GFAP or IBA1. No differences were detected in the proportion of cells labeled for either marker (*p* = 0.99; *N* = 3–4 mice per group, Fisher's exact test). This result suggests the increase in Ai14 expression observed ipsilateral to the injury was not driven by Ai14^+^ activated glia.

## Discussion

There is little question that neurons rapidly become activated in response to brain damage. Here, we applied a focal contusion injury to the somatosensory neocortex and evaluated brain cell activation around the time of injury. Consistent with prior IEG labeling studies (Dragunow and Robertson, 1988; Dragunow et al., 1990; Phillips and Belardo, 1992; Yang et al., 1994, 1995; Belluardo et al., 1995; Raghupathi et al., 1995a; Abrous et al., 1999; Förstner and Knöll, 2020), our results show focal contusion injury leads to early activation of neurons across the neocortex. Ai14^+^ neurons were observed broadly throughout the ipsilateral hemisphere, including ventral and subcortical brain areas. The area of neuron activation extended well beyond the lesion area, as determined by the presence of cortical cavity and GFAP immunostaining and remained elevated in all tissue sections examined. These observations highlight the importance of considering brain-wide effects of acute injury in neurotrauma studies.

Prior studies have reported wide variation in IEG expression after experimental brain injuries, with some studies showing IEG activation primarily near the lesion site (Dragunow and Robertson, 1988; Dragunow et al., 1990; Förstner and Knöll, 2020) and other studies reporting more widespread IEG activation (Phillips and Belardo, 1992; Yang et al., 1994, 1995; Belluardo et al., 1995; Raghupathi et al., 1995a; Abrous et al., 1999). Using a TRAP2 reporter line, which consistently labels c-*fos*–activated neurons throughout the brain, we observed broad, hemisphere-wide c-*fos* expression in a widely used focal cortical contusion model. Many cortical areas had noticeable increases in Ai14^+^ neurons, including the piriform cortex, hippocampus, entorhinal cortex, PFC, auditory cortex, and cortical amygdala; the basolateral amygdala also had overt increases in Ai14^+^ cells but was not quantified here. Therefore, a focal nonpenetrating contusion injury has potential to broadly affect the brain in the early postinjury period, even in brain areas that are not directly damaged by the mechanical insult. Support for this idea is provided by fMRI studies in rodents and humans showing broad activation of dispersed brain regions after brain injury compared with healthy controls (McAllister et al., 1999; Christodoulou et al., 2001; Harris et al., 2016).

One possibility for widespread neuron activation after TBI is the presence of injury-induced seizures, which have been reported immediately after CCI in mice (Hunt et al., 2010). However, seizures in these animals are often generalized events that would likely produce bilateral Ai14 expression (Naik et al., 2022), and seizures are only observed in a small minority of animals during the time window immediately following TBI in which neurons would be tagged by TRAP (Hunt et al., 2010). Another possibility is cortical spreading depolarization, which has long been suspected to contribute to the hemisphere-wide pattern of neuron activation after injury (Herrera and Robertson, 1996). Spreading depolarization occurs in >50% of people with TBI (Hartings et al., 2011) and in animal models (Aboghazleh et al., 2021). However, these events are rare in animal models, often involve only a single depolarization wave, and only occasionally involve the entire hemisphere in human patients (Taş et al., 2019). It is currently unknown precisely how far a spreading depolarization event might extend from the injury site in an animal model of TBI. Nonetheless, widespread network activity spanning anterior to posterior cortex has been documented in brain slices of a genetic model of epilepsy (Hou et al., 2021).

Following TBI, injured brain circuits undergo time-dependent structural and functional reorganization. This includes cell-type–specific changes in neuron morphology, intrinsic properties, and synaptic and circuit function (Hunt et al., 2013). While the cellular trigger for these plasticity responses is unknown, an emerging literature provides broad support for synaptic circuit reorganization at the level of cell ensembles. At a behavioral level, post-traumatic retrograde amnesia can be rescued by activating neurons that were tagged during the memory-forming experience prior to injury, suggesting the ensemble representing the lost memory physically exists after TBI but may be more difficult to access (Chapman et al., 2024). At a structural level, synaptic inputs are selectively gained or removed in a cell-type–specific manner (Hunt et al., 2009, 2010, 2011; Frankowski et al., 2022), and grafted inhibitory neurons are capable of reestablishing long-range connections lost to TBI (Frankowski et al., 2022), which likely underlies the prevention of epilepsy and improved memory precision after TBI (Zhu et al., 2019). While speculative, it is enticing to hypothesize that neurons activated at the time of injury may participate in an “injury cell ensemble” and function differently later in life, as compared with neurons not acutely activated by the initial injury.

Our results show there is broad neuron activation across large portions of the cortex early after a contusion injury in mice, but it is largely restricted to the ipsilateral hemisphere. These results highlight the importance of considering injury dynamics in the initial response to injury and may explain why brain areas distant from the injury become rewired even when not directly damaged by the mechanical insult.

## Synthesis

Reviewing Editor: Harald Sontheimer, University of Virginia Decisions are customarily a result of the Reviewing Editor and the peer reviewers coming together and discussing their recommendations until a consensus is reached. When revisions are invited, a fact-based synthesis statement explaining their decision and outlining what is needed to prepare a revision will be listed below. The following reviewer(s) agreed to reveal their identity: NONE.

Both referees agree on a misrepresentation of the „acuteness" of the study being misrepresented due to methodological issues. Both feels that this can be addressed by revision of the paper without additional experiments. I am copying the comments of one referee as the second one is almost identical.

In this revision, the authors attempt to define the temporal window of early gene activation that can be captured with their experimental design based on previously published work. While the language in the manuscript is now more precise regarding "early activation," the arguments provided, both in the manuscript and in the rebuttal letter, are not consistent with the literature they cite and, in some cases, directly contradict it.

In their rebuttal letter, the authors justify their experimental design by stating that the recombination window of 4-OHT is 6-12 hours and the recombination window for tamoxifen is longer than 24 hours. However, this is not supported by the literature they cite. In Guenthner 2013, recombination starts immediately after injection of 4-OHT and drops quickly, with almost no recombination at later time points such as 6-12 hours. Therefore, the claim that there is a delayed recombination window is not consistent with the data in that paper. The other reference they provide (Onishi et al. 2024) only discusses that 4-OHT has a shorter half-life (~12 hours) compared to tamoxifen, but it does not provide evidence about how fast recombination starts or supports the idea of a 6-hour delay.

The authors justify the use of tamoxifen "to ensure we capture the full extent of early activation after TBI" (lines 89-90), but based on the paper they cite, "TRAPing was maximal when stimulation occurs 23-24 hours after injection. No TRAPing above the level of the dark control occurred when light was given 6-7 h before the injection or 35-36 h after injection". In other words, they are not capturing the full extent of early activation and they mostly capture the neurons that are active 24 hours after the injury. If the goal is to capture early activation, using tamoxifen at this time point is not justified by the literature. It would largely miss early responses and instead capture later activity.

The comparison they make to Brown et al. 2022 is also misleading. In that study, tamoxifen was administered two days before the injury, meaning that by the time of the injury the drug had already been metabolized and was active in the system. This allows capturing activation starting immediately after the injury. This is fundamentally different from what the authors did here, where tamoxifen is given at or around the time of injury.

There is also a conceptual issue in the manuscript. In line 150, the authors state that the recombination window would be "~6 hr prior to and ~24 hr after the time of brain injury." It is unclear how the recombination window could start before tamoxifen administration. This does not align with the pharmacokinetics of the system.

Finally, even based only on the literature cited by the authors, within the first 12 hours very few neurons would be TRAPed using this approach (Guenthner 2013). Therefore, I do not find evidence in the literature provided by the authors that their experimental design allows them to capture early neuronal activation after TBI.

All my minor revisions still stand and I think the authors should change them before publishing:

- Authors need to do a better job at providing references. Many of them are missing (line 27, line 42, line 54...). Also, the authors mostly relied on reviews for the introduction (just to give an example, out of the 13 references of the first paragraph in the introduction, only three of them were original papers). While using reviews can be a good idea when talking about a specific point of view of the community, it is not acceptable when referring as things like "IEG are indicators of brain damage". Also, abstracts from 2013 (not peer-reviewed and not easily accessible) were provided as references instead of peer-reviewed articles. Finally, there are some important mistakes. For example, line 36 Nambu et al. is cited as Nombu et al. (even if the reference in the bibliography is correct) and Abrous is cited as Nora Abrous (line 47) but appears as Abrous in the literature, but it is placed alphabetically after Nambu (line 398). In summary, please, revise and pay attention to the references that are provided and make sure they are actually supporting the point the authors are trying to make with scientific evidence.

- Line 74: Provide AP exact coordinates relative to bregma.

- Please provide RRID numbers of the antibodies

- Please, specify the incubation time of antibodies and the blocking buffer used for this purpose.

- The first time PVT appears in the text (line 123) is not defined.

- Line 126: how was it randomly selected? Explain how the randomization happened.

- In the statistic section please specify how the normality and homoscedasticity (conditions to be able to apply ANOVA) were tested.

- In line 266 the authors define spreading polarization, which occurs to 50% of people with TBI and in animal models, as a rare event. An event that affects 50% of individuals cannot be defined as rare.
